# MEASURING NETWORK WELL-BEING AND HEALTH AMONG PEOPLE LIVING WITH DEMENTIA: A MIXED-METHODS STUDY

**DOI:** 10.1093/geroni/igz038.278

**Published:** 2019-11-08

**Authors:** Eleanor S McConnell, Sijia Wei, Bada Kang, Samantha Woog, Kayla Wright-Freeman, Kirsten N Corazzini

**Affiliations:** Duke University, Durham, North Carolina, United States

## Abstract

The feasibility and utility of measuring social networks of people living with mild to moderate stage dementia to improve care quality was examined by comparing information obtained using Antonucci’s social network mapping approach and through information elicited through a series of open-ended questions regarding life story and well-being. Data were obtained from 24 interviews with 12 people belonging to one or more networks of people living with dementia receiving care in adult day programs. Concurrently we obtained measures of health and well-being using validated symptom checklists and the ICE-CAP suite of well-being measures. Parallel interviews were conducted with social network members who were in a care partner role, either paid or unpaid. Respondents were able to map social networks, and preferred open-ended questions to more standardized measures of quality of life and well-being. Findings from both sources were generally convergent, with open-ended questions providing richer information to guide care.

